# Supplementary material for: Long-range transport of 2D excitons with acoustic waves
Source: Nat Commun. 2022 Mar 14;13:1334. doi: 10.1038/s41467-022-29042-9 (PMC8921513; doi:10.1038/s41467-022-29042-9)
Supplement: Supplementary file 1 — Supplementary Information [file 41467_2022_29042_MOESM1_ESM.docx]

**SUPPLEMENTARY INFORMATION**

**Long-range transport of 2D excitons with acoustic waves**

Ruoming Peng^1^, Adina Ripin^2^, Yusen Ye^3^, Jiayi Zhu^2^, Changming Wu^1^, Seokhyeong Lee^1^, Huan Li^1,^[[1]](#footnote-1)^^ , K. Watanabe^4^, T. Taniguchi^4^, Ting Cao^3^, Xiaodong Xu^2,3^, Mo Li^1,2, ^[[2]](#footnote-2)^^

*^1^Department of Electrical and Computer Engineering, University of Washington, Seattle, WA 98195, USA*

*^2^Department of Physics, University of Washington, Seattle, WA 98195, USA*

*^3^Department of Material Science and Engineering, University of Washington, Seattle, WA 98195, USA*

*^4^Research Center for Functional Materials, National Institute for Materials Science, Tsukuba, Japan*

1. **Theory of exciton transport driven by surface acoustic waves**

In the IXs of bilayer WSe_2_, the bound electrons and holes live, respectively, in the Q (also called Λ in literature) and the K valleys of the Brillouin zone^1–3^. Due to the spin-valley locking of bilayer WSe_2_, the dipole moment of an IX can point along the +z or -z directions, depending on the layer localization of the electron and the hole in the IX. Under the periodic out-of-plane electric field induced by the SAW, the two types of IXs with opposite dipoles are driven to two nearby field extrema separated by half acoustic wavelength and propagate together in the same direction.

In the temperature range of 6 K – 300 K, excitons have average kinetic energy of 0.6 meV – 26 meV. Taking the effective mass of an exciton to be ~ *m*_e_ (bare electron mass), the thermal velocity *v*_IX_ of the exciton is in the range of 1.5 × 10^4^ – 9.6 × 10^4^ m/s. This value is an order of magnitude lager than the travelling velocity of SAW (~3.5× 10^3^ m/s). As a result, the IX ensemble can be approximated as an exciton gas under quasi-equilibrium, when it is trapped at the minima of the slowly-varying potential energy landscape. Here the trap can arise either from disorder or from the electric field gradient induced by SAW.

We next model the transport of excitons in the realistic bilayer WSe_2_ device, assuming that the excitons do not recombine during the transport. The surface potential disorder caused by strain and other imperfections are described by potential wells that can trap excitons (Fig. SI-1 a), with a characteristic barrier height of Δ and length *L*. If *L* is much smaller than the wavelength of the SAW, the electric field gradient of SAW can help the excitons to overcome these potential barriers. An exciton that is not localized by the disorder should have kinetic energy larger than $E_{min}=\Delta-\left( \nabla E_{z-max}\cdot\mathbf{p} \right)L$ (Fig. SI-1 b) to overcome the barrier, where $\nabla E_{z-max}$ refers to the maximum gradient of the electric field induced by the SAW. Under quasi-equilibrium, the population $n$ of these excitons:

$n(\nabla E_{z-max}, T)\int_{E_{min}}^{\infty} dEe^{-\frac{E-\mu}{k_{B}T}}\cdot e^{\frac{Lp}{k_{B}T}\nabla E_{z-max}}$

The density of states factor is taken as a step function for 2D excitons.

At 100K, the PL intensity at the edge of the bilayer WSe_2_ flake should be proportional to $n$. As the maximum electric field gradient induced by SAW is proportional to the square root of SAW power $\nabla E_{z-max}\sim\sqrt{P_{s}}$, we fit the PL intensity with $n(P_{s})=n_{0}e^{\sqrt{\frac{P_{s}}{P_{t}}}}$ , where *P*_t_ is a temperature and sample-quality dependent factor, above which the exciton can respond efficiently to the electric field generated by SAW. Fig. SI-1 c shows this exponential dependence is consistent with experimental observations.

**Supplementary Figure 1.** Exciton transport by surface acoustic wave under disorder potential. (a) Schematic exciton gas in a trap with a characteristic depth in energy of $\text{∆}$ and length $L$. The exciton gas in quasi-equilibrium is shown in orange. (b) Schematic exciton gas when maximum electric potential gradient from SAW is applied, creating a shallower potential barrier on one side of the trap. The exciton population above the dotted line can travel with SAW. (c) Fitting the power-dependent PL intensity to the SAW power.


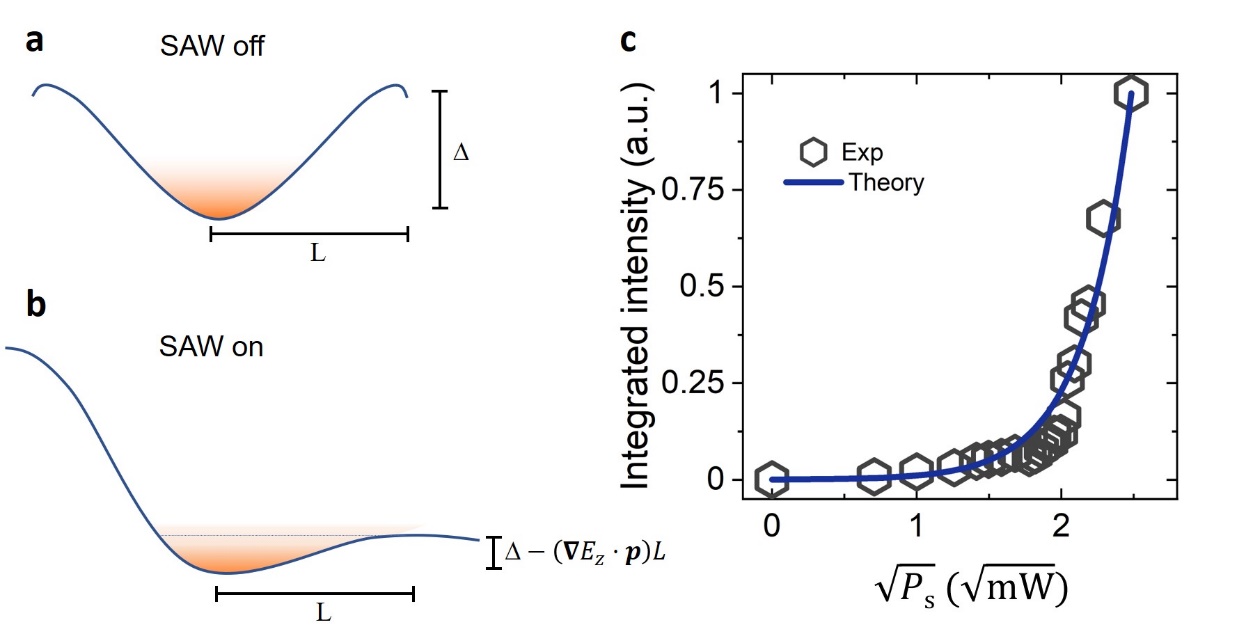


**Supplementary Figure 2.** Emission spectrum at the pump position when T=6 K. When the SAW is on, all the sharp emission resonances disappear due to delocalization of the excitons and reduced coupling with localized phonon modes.


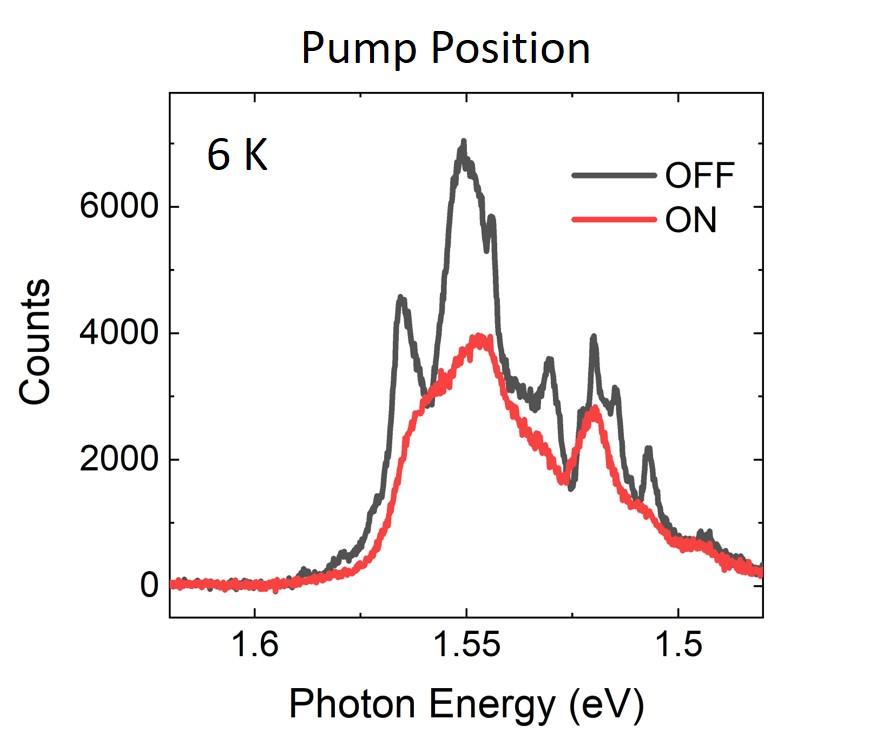


**Supplementary Figure 3.** The emission spectrum at the pump (a) and edge (b) position when T=30 K.


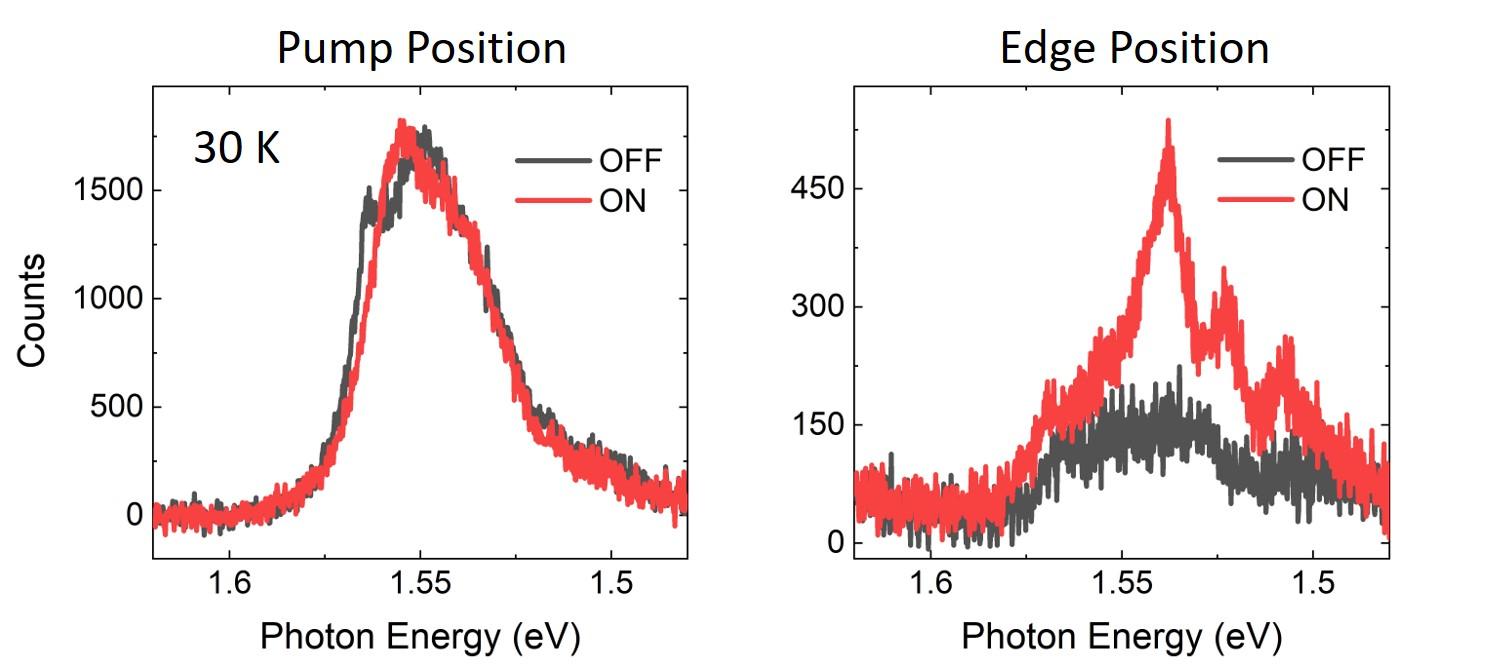


**Supplementary Figure 4.** The emission spectrum at the pump (a) and edge (b) position when T=200 K.


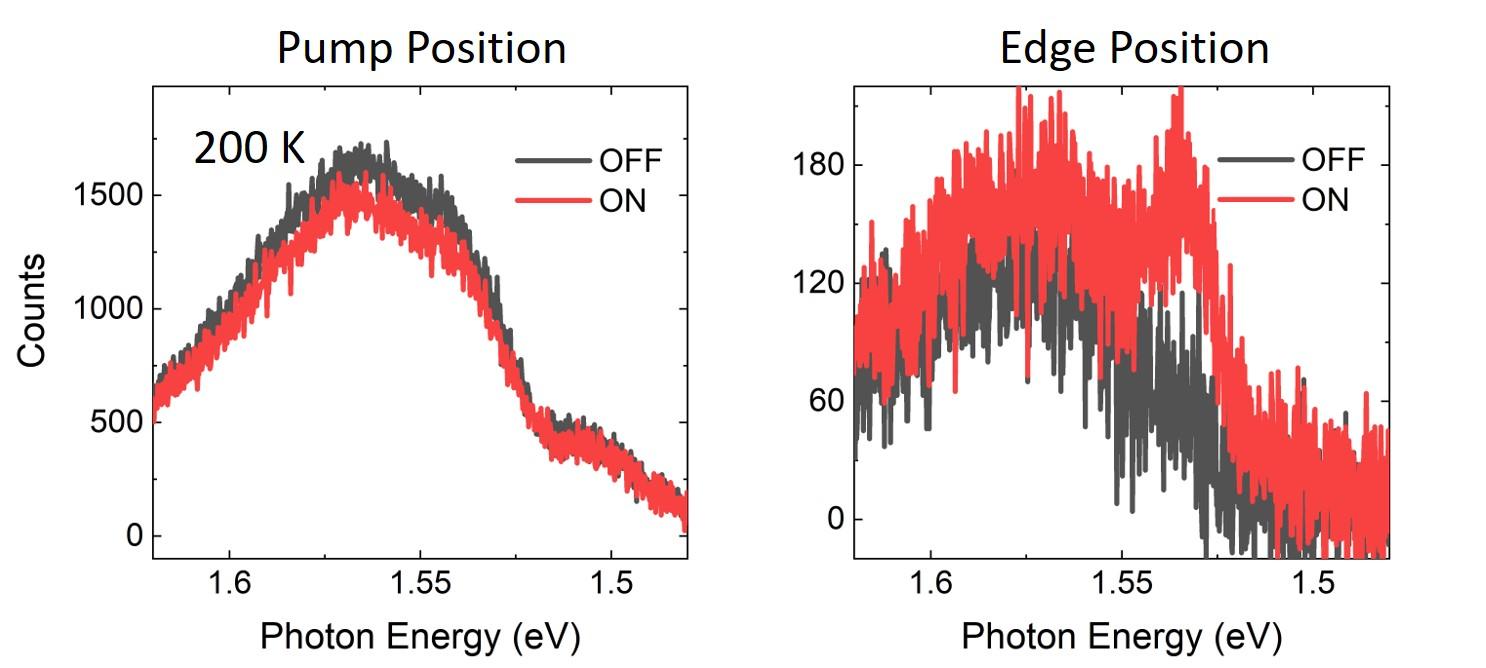


**Supplementary Figure 5.** Exciton transport at 50 K.


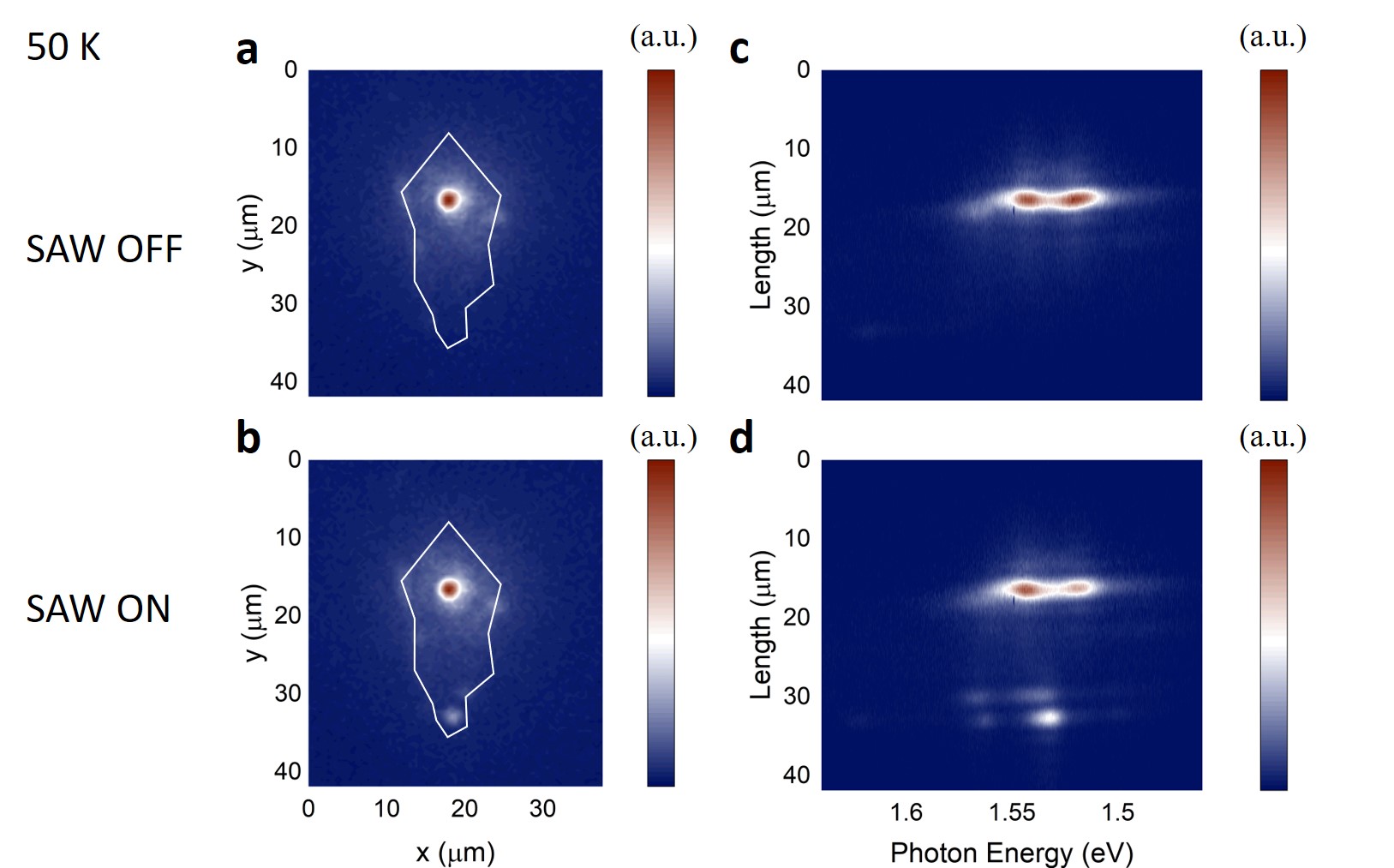


**Supplementary Figure 6.** Exciton transport at 80 K.


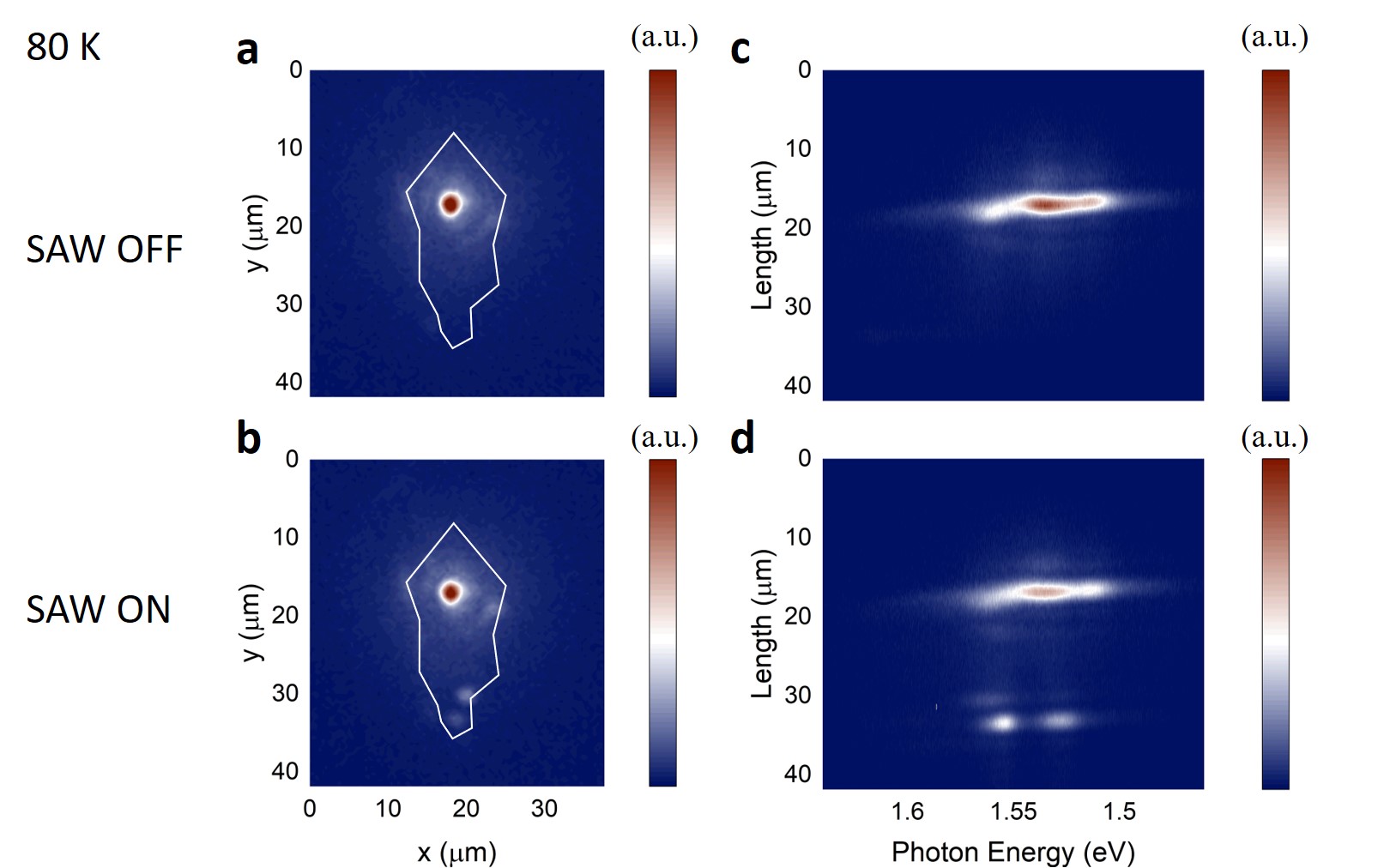


**Supplementary Figure 7.** Exciton transport at 120 K.


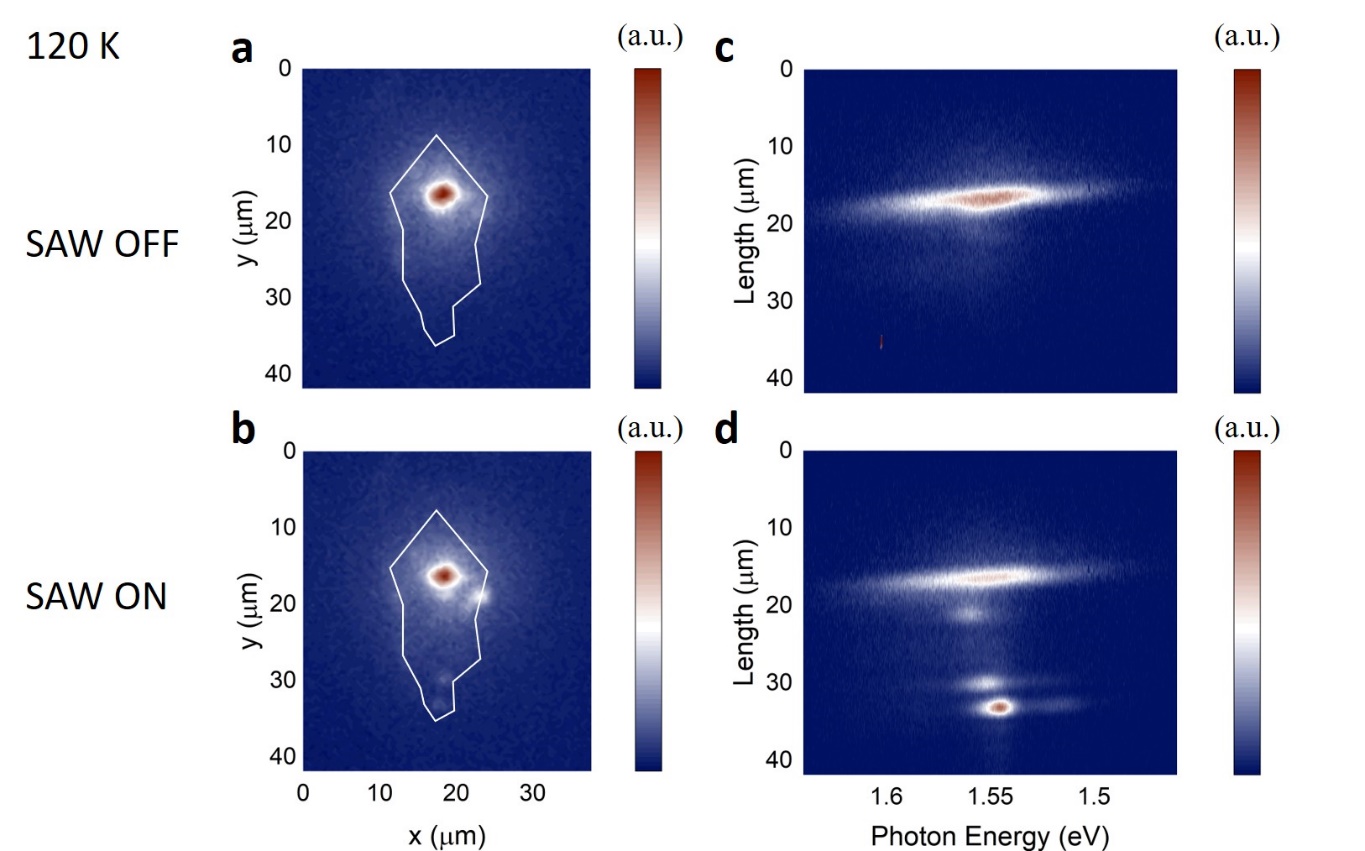


**Supplementary Figure 8.** Exciton transport at 290 K. The lower panels are zoomed in view of the dashed box. The exciton transport over ~2 µm can still be observed at room temperature owing to the strong SAW modulation.


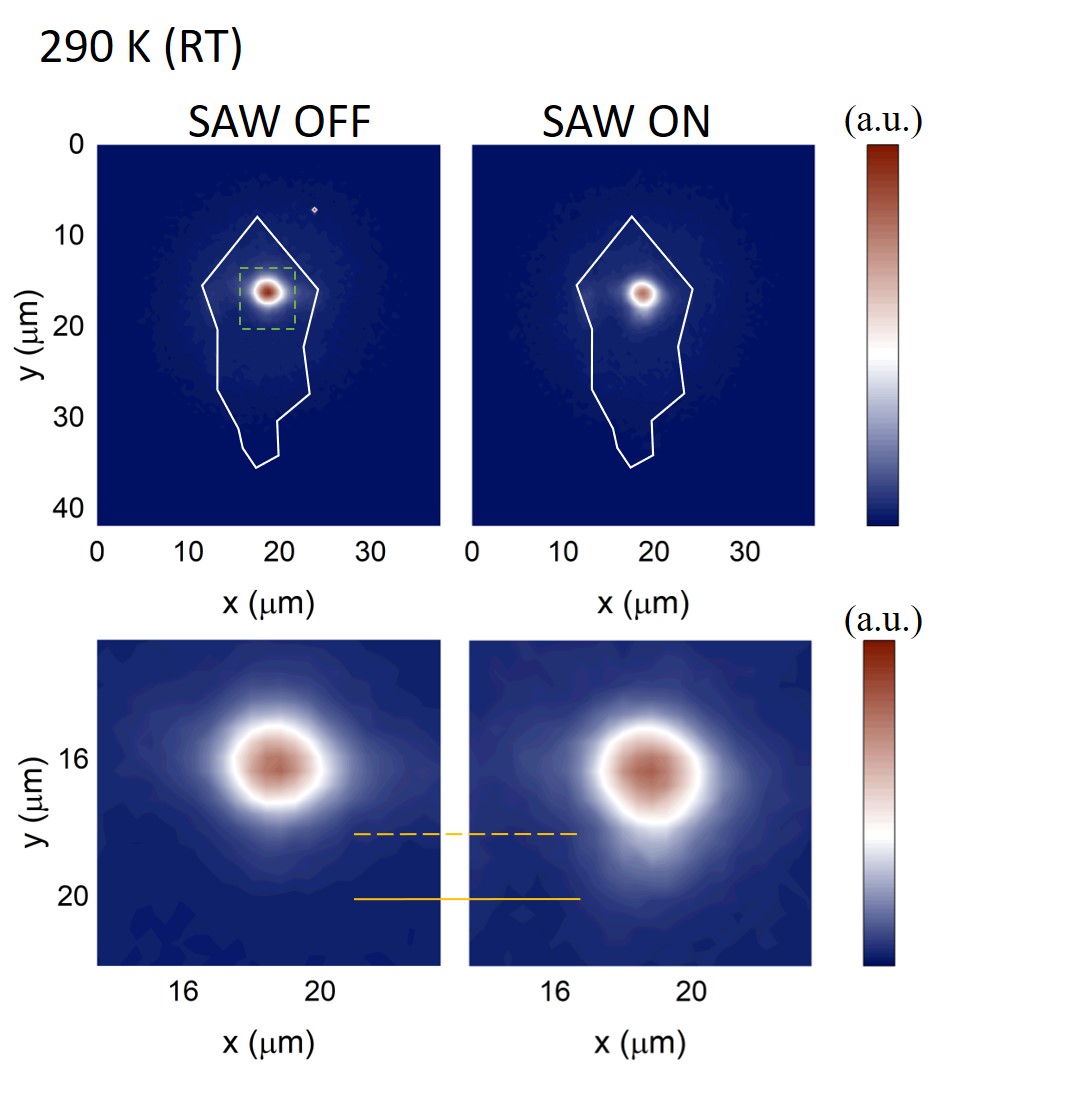


**Supplementary Figure 9.** Evaluation of the exciton transport at different temperatures. The exciton emission at the edge is normalized to the emission intensity at the pump center. The exciton transport reaches the maximum at 100 K.


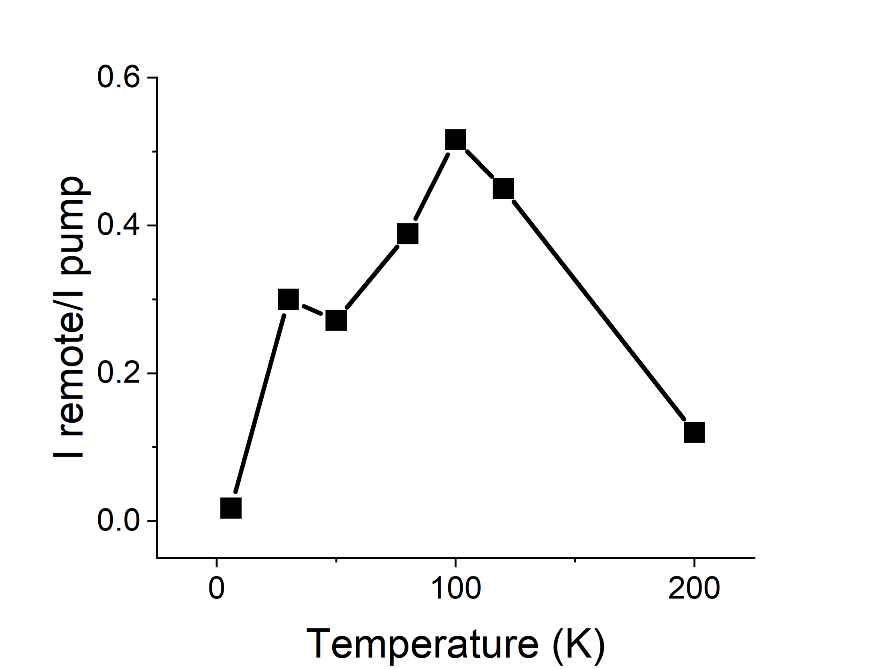


**Supplementary Figure 10.** Optical power-dependent measurement of exciton transport at 100 K with 6 mW SAW power. The portion of the transported exciton decreases with a higher optical pump. The high density of exciton could screen the piezoelectric field of SAW and reduce the acoustic modulation of the exciton energy.


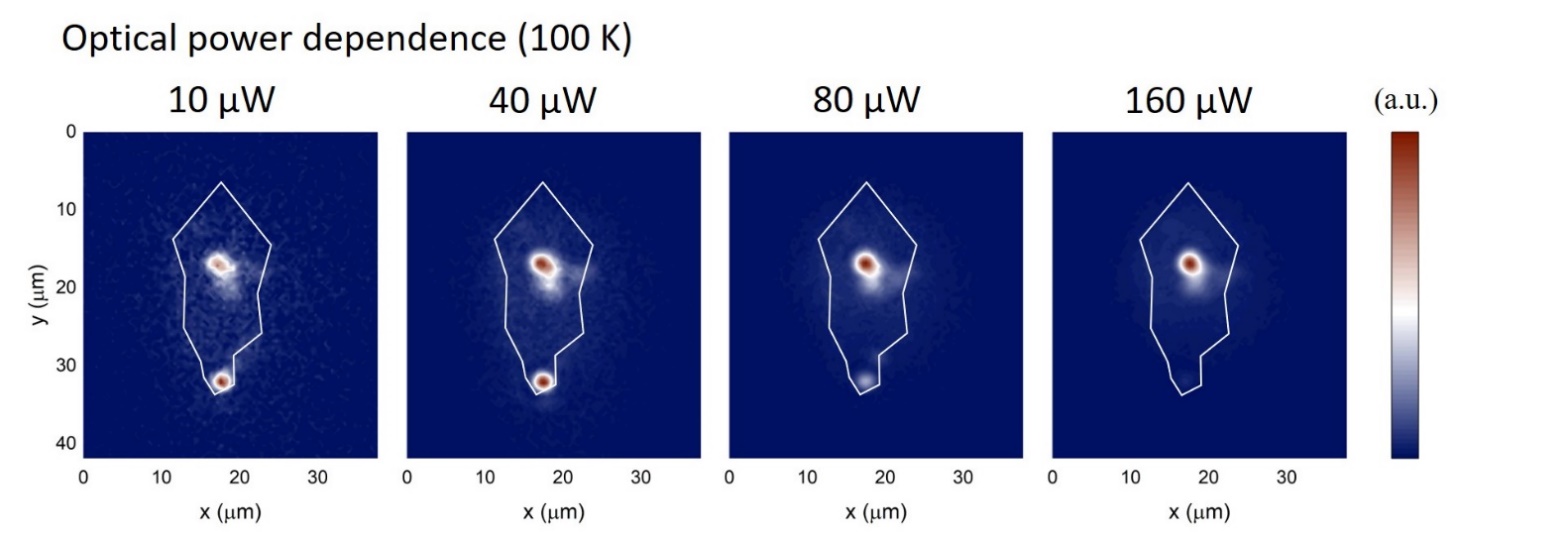


**Supplementary Figure 11.** SAW modulation of the control devices that do not have the top ITO to screen the in-plane piezoelectric field. No transport of IXs beyond the diffusion distance can be observed. Rather, the SAW piezoelectric field dissociates the IXs to free carriers to suppress the emission everywhere.


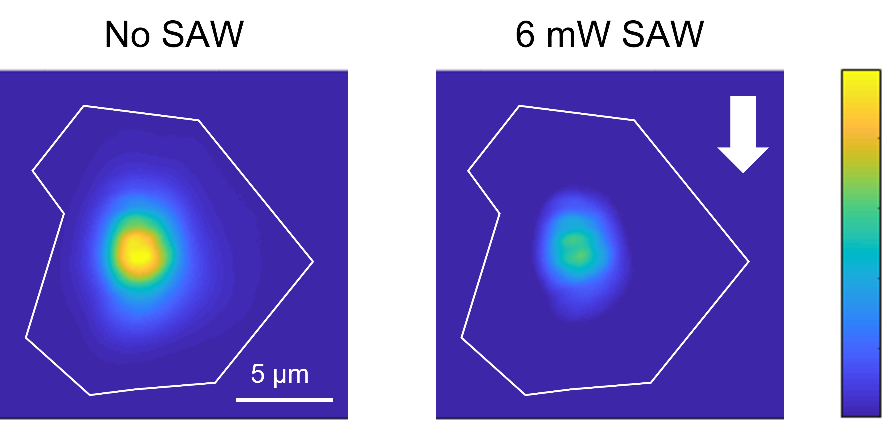


**Supplementary Figure 12.** The finite-element method (FEM) simulation of the SAW propagating on the LiNbO_3_ substrate. (a) the magnified displacement of the SAW. (b), (c) the in-plane component of the piezoelectric field with and without ITO cladding. The ITO layer can strongly screen the in-plane field.


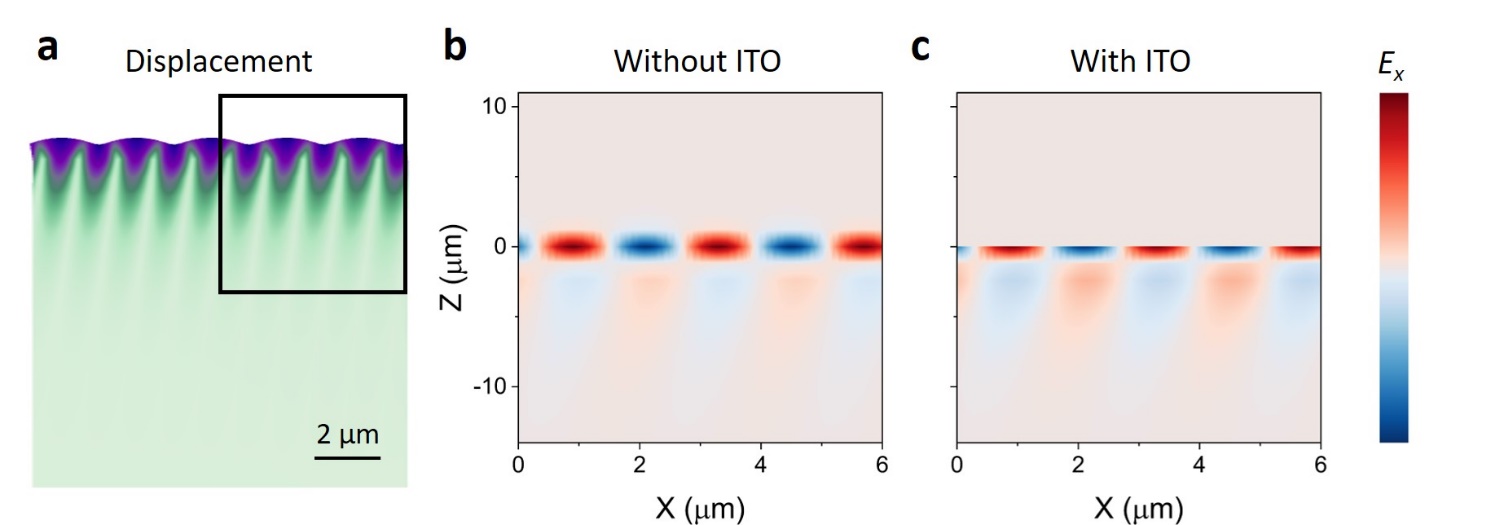


**Supplementary Figure 13.** RF calibration of the IDT acoustic transducers. (a) RF reflection coefficient S_11_ measurement of the IDT devices. We test the IDTs with varied number of periods N. Device with N=42 exhibits the best impedance matching according to the S_11_ measurements. (b) The vector analysis of the IDT resonance on a polar chart showing a strong acoustic resonance at 1.237 GHz with an on-resonance impedance 53.66 Ω+j3.77 Ω, closely matching the 50 Ω impedance of the RF source. (c) Equivalent circuit model of the IDT acoustic transducer for N=42 with component values of serial resistance *R_s_*, shunt resistance *R_l_*, capacitor *C_e_* and admittance *Y_a_* extracted from the data in (a) and (b).


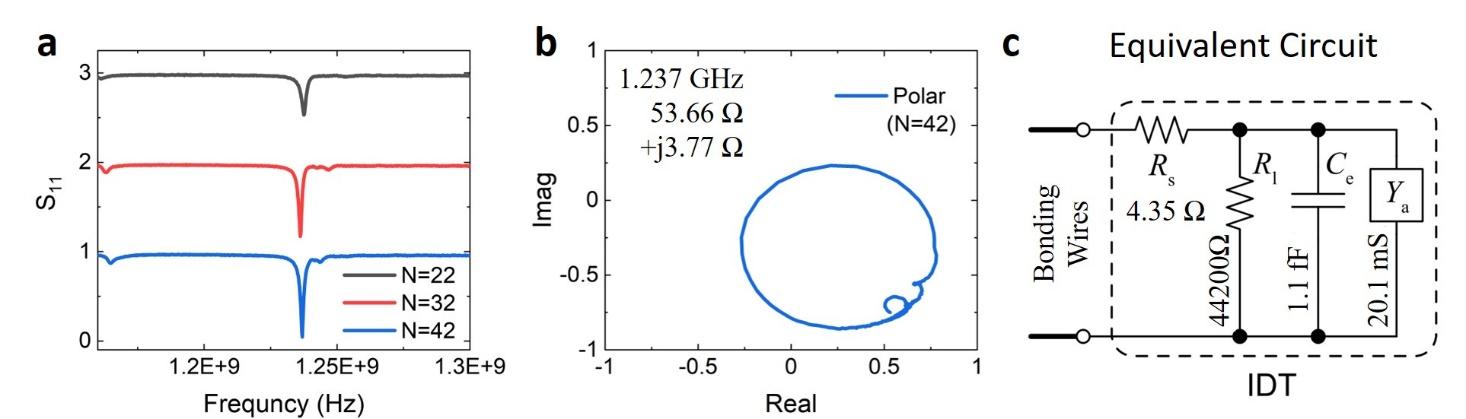


**Supplementary Figure 14.** The RF power ratio for different circuits components (Reflection, serial resistance *R_s_*, shunt resistance *R_l_* and admittance *Y_a_*) calculated from the data in Fig.SI-11 (a) and (b). For N=42, the on-resonance conversion efficiency is 91% indicating a strong RF to acoustic wave conversion.


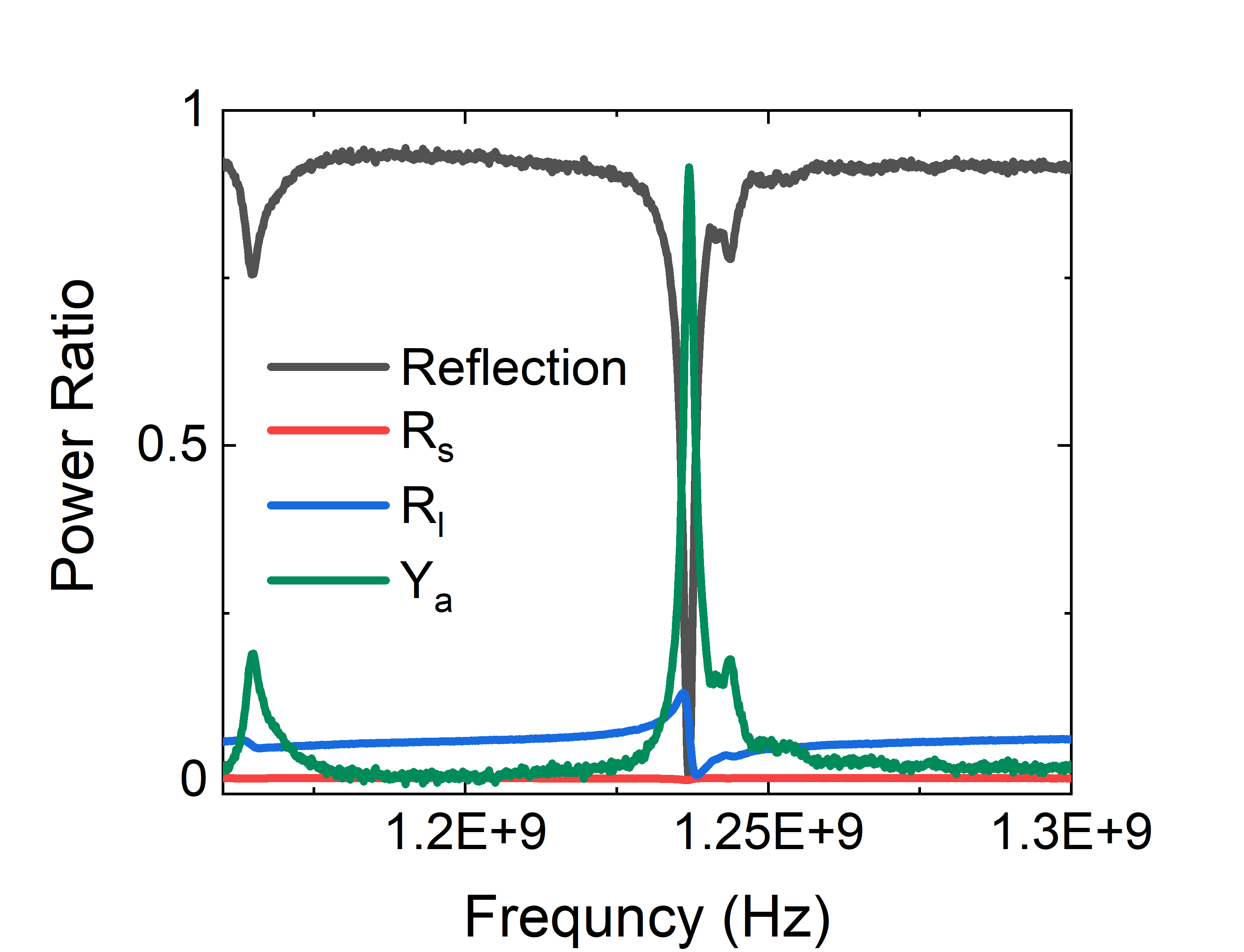


**Supplementary Table 1.** The simulated piezoelectric field at 10 nm above the LiNbO_3_ substrate with the top ITO electrode. The ITO layer can provide an efficient screening of the in-plane field component for the SAW with different wavelengths.


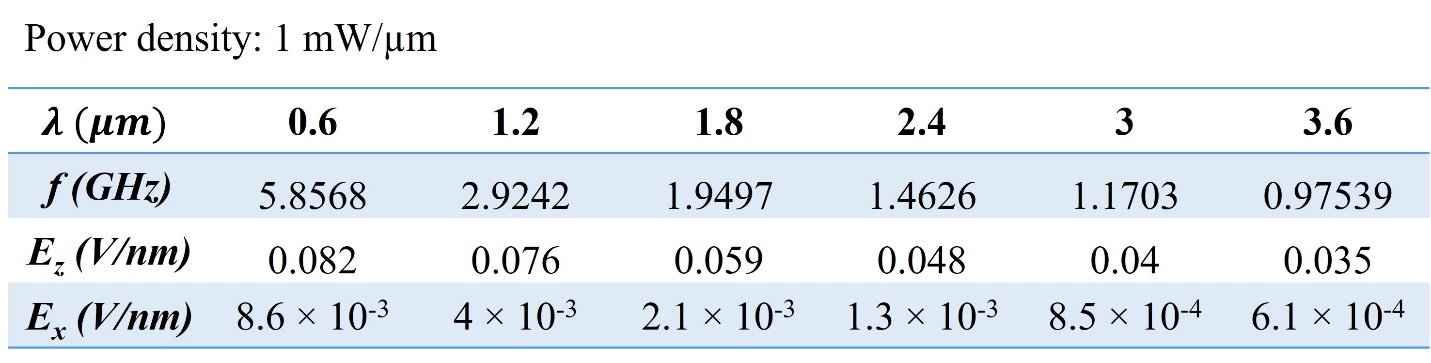


**Reference:**

1. Wilson, N. R. *et al.* Determination of band offsets, hybridization, and exciton binding in 2D semiconductor heterostructures. *Sci. Adv.* **3**, e1601832 (2017).

2. Nguyen, P. V. *et al.* Visualizing electrostatic gating effects in two-dimensional heterostructures. *Nature* **572**, 220–223 (2019).

3. Aslan, O. B., Deng, M., Brongersma, M. L. & Heinz, T. F. Strained bilayer WSe2 with reduced exciton-phonon coupling. *Phys. Rev. B* **101**, 115305 (2020).

4. Zomer, P. J., Guimarães, M. H. D., Brant, J. C., Tombros, N. & Van Wees, B. J. Fast pick up technique for high quality heterostructures of bilayer graphene and hexagonal boron nitride. *Appl. Phys. Lett.* **105**, 013101 (2014).

5. Purdie, D. G. *et al.* Cleaning interfaces in layered materials heterostructures. *Nat. Commun.* **9**, 5387 (2018).

1. Present Address: Zhejiang University, China [↑](#footnote-ref-1)
2. Email: moli96@uw.edu [↑](#footnote-ref-2)
